# Nature of association between rural background and practice location: A comparison of general practitioners and specialists

**DOI:** 10.1186/1472-6963-11-63

**Published:** 2011-03-23

**Authors:** Matthew R McGrail, John S Humphreys, Catherine M Joyce

**Affiliations:** 1 Monash University, Gippsland Medical School, Northways Road, Churchill, Victoria, 3825, Australia; 2 Monash University School of Rural Health, PO Box 666, Bendigo, Victoria, 3552, Australia; 3 Monash University, Department of Epidemiology and Preventive Medicine, 6th Floor, The Alfred Centre, Melbourne, Victoria, 3004, Australia

## Abstract

**Background:**

Rural and remote areas are characterised by a shortage of medical practitioners. Rural background has been shown to be a significant factor associated with medical graduates' intentions and decisions to practise within a rural area, though most studies have only used simple definitions of rural background and not previously looked at specialists. This paper aims to investigate in detail the nature of the association between rural background and practice location of Australian general practitioners (GPs) and specialists

**Methods:**

Data for 3156 GPs and 2425 specialists were obtained from the *Medicine in Australia: Balancing Employment and Life *(MABEL) study. Data on the number of childhood years resident in a rural location and population size of their rural childhood location were matched against current practice location. Logistic regression modelling was used to calculate adjusted associations between doctors in rural practice and rural background, sex and age.

**Results:**

GPs with at least 6 years of their childhood spent in a rural area were significantly more likely than those with 0-5 years in a rural area to be practising in a rural location (OR 2.28, 95% CI 1.69-3.08), whilst only specialists with at least 11 years rural background were significantly more likely to be practising in a rural location (OR 2.27, 95% CI 1.77-2.91). However, for doctors with a rural background, the size of the community that they grew up in was not significantly associated with the size of the community in which they currently practise. Both female GPs and female specialists are similarly much less likely to be practising in a rural location compared with males (GPs: OR 0.53, 95% CI 0.45-0.62).

**Conclusions:**

This study elucidates the association between rural background and rural practice for both GPs and specialists. It follows that increased take-up of rural practice by new graduates requires an increased selection of students with strong rural backgrounds. However, given the considerable under-representation of rural background students in medical schools and the reluctance of females to practise in rural areas, the selection of rural background students is only part of the solution to increasing the supply of rural doctors.

## Background

Rural and remote areas are characterised by a shortage of medical practitioners both in Australia and internationally [1-4]. This shortage reflects an insufficient supply of medical graduates, ongoing recruitment and retention difficulties, changing preferences of both recent graduates and established doctors, and a prevailing negative view of both rural practice and general practice [5-8]. In the quest to overcome this rural medical workforce shortage, the Australian government has significantly increased the number of medical students in training (including those from a rural background), devolved medical education away from capital cities, and increased students' rural exposure through the establishment of Rural Clinical Schools and University Departments of Rural Health [9-15].

Many factors contribute to explaining doctors' preferences to practise in specific locations. Rural background has been shown in many different countries to be a significant factor associated with medical graduates' intentions and decisions to practise within a rural area [16-21]. A recent review of healthcare professionals found that rural background was the factor most strongly associated with rural practice [22], though this finding does not always take account of the additional impact of other factors or strategies [22,23]. The strength of association (odds ratio) between rural background and rural practice was estimated in a 2003 systematic review of 12 studies at approximately 2.0-2.5 [24]. Several more recent studies provide consistent support for this association [19,25,26].

However, most of these studies have been small in size and arguably somewhat simplistic in their assessment of the association between rural background and rural practice. The actual nature of rural background is not typically distinguished according to the specific geographical location or duration of time spent there. Furthermore, the association for specialists compared with general practitioners (GPs) has not previously been investigated. The aim of this paper, therefore, is to investigate in more detail the nature of the association between rural background and practice location of Australian doctors. Specifically it investigates, for both GPs and specialists, (i) what influence the number of childhood years resident in a rural location and population size of the main childhood rural residential location have on current practice in a rural location, and (ii) the strength of association between rural background and current rural practice, after adjusting for covariates. Additionally for specialists, the strength of association between rural background and providing rural visiting services is examined.

## Methods

This paper uses data from the *Medicine in Australia: Balancing Employment and Life *(MABEL) study, a large prospective cohort (longitudinal) study of Australian doctors (see https://mabel.org.au/). The primary aim of MABEL is to investigate labour supply decisions and their determinants among Australian doctors, and is currently funded for four annual waves. The first wave of data (n = 10,498; response rate = 19.36%), used here, was self-selected from the entire Australian medical workforce (GPs and GP registrars, specialists, specialists in training, and hospital non-specialists) between June and November 2008. Each doctor-type used a similar questionnaire, consisting of eight sections, which can be viewed at the study's website. Further details of the study protocol and baseline data have been reported elsewhere [27]. Non-response bias analysis of wave 1 found no major concerns including that older doctors were slightly under-represented, females were over-represented by six-percentage points, GPs were under-represented by four percentage points, specialists were over-represented by five percentage points, rural doctors were over-represented by four percentage points whilst hours worked was similarly distributed to the population. The study was approved by the University of Melbourne Faculty of Economics and Commerce Human Ethics Advisory Group (Ref. 0709559) and the Monash University Standing Committee on Ethics in Research Involving Humans (Ref. CF07/1102 - 2007000291).

For this paper, respondents within our study cohort were limited to doctors considered to be unrestricted in their choice of practice location. For GPs, unrestricted refers to those who are either not training as a registrar or those not mandated to practise in 'areas of need' or 'workforce shortage' such as for many International Medical Graduates [28]. For specialists, unrestricted refers to specialties that are not limited by location to practising in large metropolitan centres, specifically anaesthesia (non-ICU), diagnostic radiology, emergency medicine, general medicine, general surgery, obstetrics and gynaecology, orthopaedics, paediatrics and psychiatry. For the nine specialties included, all had at least 15% of respondents in our cohort located in cities of <100,000 residents and generally require a smaller population catchment to maintain a viable service [29].

Nature of rural background, the key predictor used in this paper, was measured using two items. The first was the number of years resident in a rural area up until school leaving age (0-18 years). Other studies have highlighted the non-linear association between rural childhood years and practice location, so we separated the number of years into four groups: 0 years, 1-5 years, 6-10 years and 11-18 years. Furthermore, rural background (duration) was separated as a binary variable by those who spent at least six years of their childhood in a rural location. The second item was the main residential rural location (if applicable) until school leaving age, which was geocoded and analysed using six community sizes: metropolitan = > = 1,000,000 residents; large regional = 100,000- 99,999 residents; large rural = 25,000-99,999 residents; medium rural = 10,000-24,999 residents; small rural = 2500 - 9999 residents; and very small rural = <2500 residents. Rural background (community size), when applied as a binary variable, was defined as communities with <100,000 residents. Current practice location (community size) was also separated into the same six categories, and dichotomised as "rural practice" for work locations with <100,000 residents.

Bivariate associations were tested using the Pearson chi-squared test. Strength of association was measured using the odds ratio (OR), together with 95% confidence intervals. Crude odds ratios are reported to enable comparison with covariate-adjusted odds ratios, calculated through logistic regression models. The two key demographic covariates included in the adjusted model were gender and age (under 40, 40-49, 50-59 and 60+). Interaction effects were tested in the adjusted models but these were not statistically significant. Deviation contrasts, which enable the comparison of each category to the unweighted average of all categories for that variable, were used for the four age groups because there is no obvious comparison group. Calculations were performed using PASW Statistics 18.0 (SPSS Inc, Chicago, Ill, USA) and StataSE 10 (StataCorp, Texas, USA).

One additional association of rural background and rural practice was assessed for specialists who provide visiting services (e.g. weekly, monthly) in other geographic areas (usually smaller rural towns). For this item, all specialists (both restricted and unrestricted) were included.

## Results

Responses were received in Wave 1 from 3906 GPs (including registrars) and 4596 specialists (completed specialty training). Of these, 3156 GPs and 2425 specialists are unrestricted in location of practice. It is seen in Table 1 that doctors with some rural background make up between 20% and 25% of the MABEL Wave 1 cohort. With Australia having around 35% of its population residing in rural areas, this highlights the historical under-representation of medical students from rural backgrounds, despite government initiatives designed to increase their proportion [12]. Only minor differences are seen by gender and by age for both GPs and specialists, though there was a significant association between age and rural background for specialists (p = 0.02). Additionally, GPs and specialists are equally likely to have some rural background (p = 0.44).

**Table 1:** Characteristics of respondents (unrestricted GPs and Specialists)

	GP Background*		Specialist Background*	
	Rural	Metropolitan	Rural	Metropolitan
**Total**	711 (23.1%)	2369 (76.9%)	525 (22.2%)	1839 (77.8%)
**Males**	411 (24.1%)	1295 (75.9%)	375 (22.1%)	1323 (77.9%)
**Females**	300 (21.8%)	1074 (78.2%)	150 (22.5%)	516 (77.5%)
**Under 40**	91 (20.8%)	347 (79.2%)	82 (22.6%)	281 (77.4%)
**40-49**	211 (22.5%)	725 (77.5%)	148 (18.9%)	637 (81.1%)
**50-59**	274 (23.7%)	884 (76.3%)	166 (23.2%)	551 (76.8%)
**60+**	135 (24.6%)	413 (75.4%)	129 (26.1%)	366 (73.9%)

*Background location missing for 76 GPs and 61 specialists; Age missing for 4 specialists

### Associations between nature of rural background and practice location

Table 2 shows the association between rural background (both duration and location) and current place of practice for both GPs and specialists. There is a clear association between the number of childhood years resident in a rural location and subsequently practising in a rural location (p < 0.001). Only 28% of GPs with no childhood years spent in a rural location and 32% of GPs with 1-5 childhood years in a rural location are currently practising in a rural area. This rises considerably to 47% for GPs with 6-10 childhood years in a rural location and 48% of GPs with more than 10 childhood years in a rural location currently practising in a rural area. Closer investigation reveals that GPs with either no rural childhood years or 1-5 rural childhood years are similar in their distribution of current practice location, whilst GPs with 6-10 and 11-18 rural childhood years are also similarly distributed.

**Table 2:** Distribution of Australian GPs' and specialists' current practice location (community size) by number of childhood years in a rural location and size of childhood rural location

Childhood rural background	Current Practice Location (Community Size)*					
	Metropolitan	Large regional	Large rural	Medium rural	Small rural	Very small rural
GPs (n)*	1716	341	318	214	274	204
**0 years (70%)**	61%	11%	9%	7%	7%	5%
**1-5 years (7%)**	58%	10%	9%	10%	7%	6%
**6-10 years (6%)**	44%	9%	14%	7%	14%	12%
**11-18 years (17%)**	39%	13%	17%	8%	13%	10%
**Large/medium rural (10%)**	43%	12%	18%	10%	8%	9%
**Small/very small rural (13%)**	43%	11%	14%	7%	15%	10%
						
Specialists (n)*	1434	527	288	139	26	-
**0 years (73%)**	62%	22%	10%	5%	1%	-
**1-5 years (5%)**	63%	21%	12%	2%	2%	-
**6-10 years (5%)**	55%	27%	13%	3%	2%	-
**11-18 years (17%)**	48%	22%	19%	10%	1%	-
**Large/medium rural (9%)**	54%	19%	19%	6%	2%	-
**Small/very small rural (11%)**	56%	22%	15%	6%	1%	-

* Current practice location missing for 14 GPs and 11 specialists; Rural childhood years missing for 75 GPs and 54 specialists

Doctors who grew up in a metropolitan or large regional location (GPs - 77%, specialists - 80%) are distributed near identically to those with 0 years rural background

For specialists, minimal difference was seen in the distribution of current practice location for those with 0 years (16% practising in a rural area), 1-5 years (16% practice rural) and 6-10 childhood years in a rural location (18% practice rural). However, the number of specialists practising in a rural location rises dramatically to 30% for those with 11-18 childhood years spent in a rural location (p < 0.001).

For GPs and specialists who spent some of their childhood in a rural location, there appears to be only a minimal association between the size of the childhood community and the size of their current practice location. Unlike the number of childhood years resident in a rural location, size of childhood community appears to have little bearing on the size of practice location community once below 100,000 residents. Doctors who grew up in rural communities ranging from large rural towns through to very small rural towns are not more or less likely to be practising in similar sized communities.

### Strength of association for GPs between rural background, covariates, and rural practice

Table 3 shows for GPs the strength of association between rural background and practising in a rural area (communities <100,000 residents). Similar to Table 2 results, there is no statistically significant difference for GPs between those with 1-5 years and 0 years of rural childhood both for crude association (OR 1.21, 95% CI 0.89-1.63) and adjusted association (OR 1.20, 0.89-1.63) with rural practice. However, those with both 6-10 years and 11-18 childhood rural years were equally statistically significantly associated with rural practice both for crude associations (OR 2.30, 95% CI 1.70-3.10 and OR 2.32, 95% CI 1.91-2.83) and adjusted associations (OR 2.28, 95% CI 1.69-3.08 and OR 2.35, 95% CI 1.93-2.87).

**Table 3:** Strength of association between growing up in a rural location and practising in a rural location for Australian GPs

Unrestricted GPs	Rural Background	Rural Practice (%)	Crude Odds Ratio (95% CI)	Adjusted Odds Ratio (95% CI)
**Childhood years resident in a rural area (0 years reference group)**	0 years	28.2%	-	-
	1-5 years	32.1%	1.21 (0.89, 1.63)	1.20 (0.89-1.63)
	6-10 years	47.4%	2.30 (1.70, 3.10)	2.28 (1.69-3.08)
	11-18 years	47.7%	2.32 (1.91, 2.83)	2.35 (1.93-2.87)
**Male (55.3%)**	Rural -24.1%	55.3%	2.52 (2.01, 3.17)	1.89 (1.60-2.23)
	Metro - 75.9%	32.9%		
**Female (44.7%)**	Rural -21.8%	37.1%	1.94 (1.47, 2.55)	0.53 (0.45-0.62)
	Metro - 78.2%	23.3%		
**Under 40 (14.0%)**	Rural -20.8%	51.6%	2.62 (1.63, 4.20)	1.22 (1.03-1.45)
	Metro - 79.2%	29.0%		
**40-49 (30.3%)**	Rural -22.5%	49.8%	2.52 (1.84, 3.45)	1.09 (0.96-1.24)
	Metro - 77.5%	28.2%		
**50-59 (37.6%)**	Rural -23.7%	47.3%	2.10 (1.59, 2.78)	1.03 (0.91-1.16)
	Metro - 76.3%	29.9%		
**60 and over (18.1%)**	Rural -24.6%	42.1%	2.09 (1.39, 3.15)	0.73 (0.62-0.86)
	Metro - 75.4%	25.8%		

While it appears that the crude association is somewhat similar for males and females (OR 2.52 and 1.94), there are considerably fewer females than males from both rural and metropolitan backgrounds choosing to practise in a rural area. This is reflected in the adjusted associations, with males nearly twice the odds of females to be practising in a rural area (OR 1.89, 95% CI 1.60-2.23). Younger GPs with a rural background are the most likely age cohort to be currently practising in a rural area (OR 1.22, 95% CI 1.03-1.45) while the oldest group of GPs is the least likely to be practising in a rural area (OR 0.73, 95% CI 0.62-0.86).

### Strength of association for specialists between rural background, covariates, and rural practice

Table 4 shows for specialists the association between rural background and practising in a rural area (communities <100,000 residents). As with Table 2 results, it is seen that only those with 11-18 rural childhood years had a greater likelihood of rural specialist practice (OR 2.24, 95% CI 1.75 - 2.87 and adjusted OR 2.27, 95% CI 1.77 - 2.91). There is no statistically significant difference between those practising in rural areas for specialists with 0, 1-5 or 6-10 years rural childhood years.

**Table 4:** Strength of association between growing up in a rural location and practicing in a rural location for Australian specialists

Unrestricted specialists	Rural Background	Rural Practice (%)	Crude Odds Ratio (95% CI)	Adjusted Odds Ratio (95% CI)
**Childhood years resident in a rural area (0 years reference group)**	0 years	16.2%	-	-
	1-5 years	15.9%	0.98 (0.60, 1.60)	1.02 (0.62-1.69)
	6-10 years	18.1%	1.15 (0.70, 1.87)	1.17 (0.72-1.92)
	11-18 years	30.1%	2.24 (1.75, 2.87)	2.27 (1.77-2.91)
**Male (72.0%)**	Rural -22.1%	31.3%	2.09 (1.61, 2.71)	1.75 (1.34-2.27)
	Metro -77.9%	17.9%		
**Female (28.0%)**	Rural -22.5%	18.0%	1.66 (1.01, 2.73)	0.57 (0.44-0.74)
	Metro -77.5%	11.7%		
**Under 40 (15.2%)**	Rural -22.6%	17.1%	1.24 (0.64, 2.41)	0.85 (0.67-1.08)
	Metro -77.4%	14.2%		
**40-49 (33.0%)**	Rural -18.9%	27.7%	2.01 (1.32, 3.05)	1.06 (0.89-1.26)
	Metro -81.1%	16.0%		
**50-59 (30.4%)**	Rural -23.2%	33.1%	2.49 (1.68, 3.70)	1.12 (0.94-1.33)
	Metro -76.8%	16.6%		
**60 and over (21.4%)**	Rural -26.1%	26.6%	1.72 (1.07, 2.77)	0.98 (0.81-1.20)
	Metro -73.9%	17.4%		
**Provide rural visiting service (37.7%)**	Rural -21.8%	34.3%	2.58 (1.97, 3.38)	N/A
	Metro -78.2%	16.8%		

The crude association between rural background and practising in a rural area is similar for male and female specialists (OR 2.09 and 1.66), but there is a statistically significant decrease in females from both rural and metropolitan backgrounds practising as specialists in a rural area. Again, this is reflected in the adjusted OR, with males close to twice the odds of females to be practising in a rural area (OR 1.75, 95% CI 1.34-2.27). The youngest specialists (< 40) from both backgrounds are the least likely age cohort to be practising in a rural location (OR 0.85, 95% CI 0.67 - 1.08), whilst 50-59 year old specialists from a rural background are the most likely to be practising in a rural location (OR 1.12, 95% CI 0.94 - 1.33). The final row in Table 4 shows that specialists who provide visiting services to those rural locations which are unable to support a more permanent presence are clearly more likely to come from a rural background (OR 2.58, 95% CI 1.97 - 3.38).

## Discussion

The results from this study confirm existing evidence of the strong association between childhood rural background and taking up medical practice in a rural area. Our study has demonstrated that this association applies not just to GPs but also specialists. Importantly too, for the first time, this study investigates whether there is any association between length of time spent as a child in a rural location and the likelihood of taking up rural practice, and whether the size of the childhood community relates to that in which practice is taken up.

Examination of the association between the number of rural childhood years and rural practice reveals a clear separation point, though this was different for GPs and specialists. For GPs, those with at least 6 years of their childhood in a rural location were statistically significantly more likely to practise in a rural area than those with 0-5 years of their childhood resident in a rural location. Interestingly, for specialists it was found that only those with over 10 years of their childhood in a rural location were statistically significantly more likely to practise in a rural area. Unfortunately, our study is not able to distinguish between years of rural childhood into secondary school and primary school periods, though a separate Australian study found no difference in their association with rural practice for GPs [18]. In contrast, there does not appear to be any association between the size of the community that rural background doctors spent their childhood and taking up rural practice. Doctors who grew up in communities ranging from very small rural communities through to large rural towns are equally likely to be practising as rural doctors.

All existing evidence on the association between rural background and rural practice is for GPs only. Our study has demonstrated new evidence of this association applying also to specialists. After limiting to unrestricted (by location choice) specialists, it was found that only those with at least 10 years of their childhood in a rural location were strongly associated with rural practice. The ratio between male and female specialists practising in a rural area was very similar compared to GPs. However, age and rural practice was distributed differently for specialists, with those aged 50-59 most likely to be in rural practice, whilst those aged less than 40 was the least likely to be in rural practice. This may reflect a preference for specialists to remain within larger and more proximate networks early in their career before they are ready to embark on rural practice.

It follows that increasing the proportion of medical graduates taking up rural practice requires significantly increased priority to students who have spent some proportion of their childhood in a rural location. Unfortunately, however, evidence suggests that this is difficult to achieve since rural background students have been shown to be highly less likely to apply for entry to medical school [30,31]. Rural background students seeking to enter medical school face considerable impediments, in the form of increased educational costs and, in the absence of preferred rural entry schemes, and in reduced likelihood of gaining selection based solely on academic performance achievements. Overcoming these barriers necessitates ongoing support from both government and medical schools in the form of scholarships and preferred entry for rural background students [32,33].

With the increasing feminisation of the medical workforce, currently sitting around 55% of medical students in Australia [15], the statistically significant negative association between female GPs and female specialists and take-up of rural practice, compared to their male peers, is a concern for future rural medical workforce supply [34]. This does not appear to be related to differences in rural background because it is seen that females compared to males have very similar crude associations between rural background and rural practice. However, females from both rural and urban backgrounds who practise as GPs or specialists are much less likely than males to practise in rural areas. Clearly there is scope to increase the rural workforce supply by increasing the take-up of rural practice by females to rates similar to those of males. However, this is unlikely unless expressed concerns of female doctors about difficulty in working part-time, on-call demands and more generally achieving a work-life balance are addressed [35,36].

Many rural locations are either too small or too isolated to support resident specialists, such as in cardiology, endocrinology, gastroenterology, oncology or urology. Additionally, there has been a large decline in procedural GPs in recent years [37]. For these reasons, visiting specialists often provide the only local access to available specialist services, something which is particularly important for those residents unable to travel to services in large metropolitan areas. Our results show that specialists from a rural background are twice as likely as urban background specialists to provide these important visiting or out-reach services.

A strength of our study is its large cohort size, however, this must be tempered by our low response rate of around 19% which raises concerns about the generalisability of our results. The representativeness of our cohort has been detailed elsewhere [27], but rural background was not considered in that analysis, thus the level of selection bias specific to this paper is unclear. Whilst we believe that we have optimally used rural background given the data available, it is acknowledged that there are many possible alternative measures of rural background, which were not collected in this study. Some of these include the separation of childhood years into schooling tertiles (ages 0-6, 7-12, and 13-18) and information on their spouse's rural background.

Whilst most emphasis in this study is on the strong association between rural background and rural practice, it follows that, largely due to the proportionally small number of rural background medical students, over two-thirds of all doctors currently working in rural areas do not have a rural background (Table 2 data). With the large increase in Australian medical students undertaking more training in rural communities [10,38], as well as increasing opportunities for regional-based postgraduate training [39,40], it is hoped that increased proportions of urban-background students might also be attracted to take up rural practice and thereby add to the supply emanating from rural background doctors. Selection of rural background students is just one part of the solution to increasing the supply of rural doctors.

## Conclusions

The shortage of rural medical practitioners remains a critical problem with no apparent easy solutions. Undoubtedly training medical students from a rural background plays an important part in the supply of rural doctors. This study confirms that the influence of rural background on where unrestricted doctors choose to practise is similar for GPs and specialists. There does not appear to be any statistically significant difference in practice location for doctors who grew up in different sized communities from very small rural through to large rural, but GPs with 6-18 years and specialists with 11-18 childhood years resident in a rural location are statistically significantly more likely to be practising as rural doctors. With the majority of medical students being female, their reluctance to practise in rural areas will place further pressure on the future supply of rural doctors if measures are not put in place to alleviate their concerns.

## Competing interests

The authors declare that they have no competing interests.

## Authors' contributions

MRM was responsible for analysis and interpretation of data in this study and all drafting of this paper. JSH was a chief investigator and contributed to the conception and design of this study, interpretation of results and all drafting of this paper. CMJ was a chief investigator and contributed to the conception and design of this study and interpretation of results. All authors contributed to revisions of the paper and approved the final manuscript.

## Pre-publication history

The pre-publication history for this paper can be accessed here:

http://www.biomedcentral.com/1472-6963/11/63/prepub
